# Alterations in plasma cytokine profiles in generalized myasthenia gravis following different immunotherapeutic regimens

**DOI:** 10.3389/fneur.2025.1728767

**Published:** 2025-12-05

**Authors:** Zhuangzhuang Ren, Xihui Ma, Yudan Liu, Yufeng Zhang, Yuping Chen, Chenjing Sun, Feng Qiu

**Affiliations:** 1 Department of Neurology, Chinese PLA Hospital, Beijing, China; 2 Department of Pulmonary and Critical Care Medicine, Respiratory Research Institute, The Eighth Medical Center of PLA General Hospital, Beijing, China

**Keywords:** generalized myasthenia gravis, glucocorticoids, tacrolimus, rituximab, cytokines

## Abstract

**Background:**

The pathogenesis of myasthenia gravis (MG) involves an imbalance of various pro-inflammatory and anti-inflammatory cytokines. Currently, there are no clinical studies comparing the effects of hormones and different non-hormonal immunosuppressants on the regulation of plasma cytokines. Hence, there is no unified standard for drug selection. This study aims to explore the associations between glucocorticoid (GC), tacrolimus (TAC), and rituximab (RTX) and plasma cytokine levels in generalized myasthenia gravis, providing an observational overview of how different treatment regimens relate to immune-inflammatory patterns in clinical practice.

**Methods:**

A retrospective collection of 65 GMG patients diagnosed at the Department of Neurology, General Hospital of the People’s Liberation Army from July 2022 to May 2024 was conducted. According to treatment regimens, patients were divided into a GC group (*n* = 17), a TAC group (*n* = 17), a RTX group (*n* = 9), and non-medicated patients (NM) group (*n* = 22), another 30 healthy individuals were selected as the healthy control (HC) group. Clinical data and levels of cytokines such as Interleukin (IL)-1β, IL-2, IL-4, IL-5, IL-6, IL-8, IL-10, IL-12P70, IL-17, Tumor Necrosis Factor (TNF)-*α*, Interferon (IFN)-α, and IFN-*γ* in plasma were collected to compare plasma cytokine levels across the three treatment groups and the non-medicated group.

**Results:**

Compared with the HC group, GMG patients in the NM group showed significantly higher plasma levels of IL-1β, IL-2, IL-4, IL-10, IL-17, TNF-*α*, IFN-*α*, and IFN-*γ*. Relative to the NM group, the GC group exhibited lower levels of IL-1β, IL-4, IL-6, IL-17, and TNF-α, and the RTX group showed lower levels of IL-1β, IL-4, IL-6, IFN-*α*, and TNF-α. In the comparison across treatment groups, the GC group presented lower IL-17 and IFN-*γ* levels than the TAC group, while the RTX group presented lower IL-6, IFN-α, and TNF-α levels than the TAC group.

**Conclusion:**

Cytokine levels were markedly elevated in the plasma of patients with GMG, indicating their potential involvement in disease-related immune dysregulation. Distinct treatment groups displayed different cytokine patterns, reflecting heterogeneous immunological states among patients receiving GC, TAC, or RTX. Collectively, these findings provide preliminary immunological insights into GMG and highlight the need for validation in larger, prospective studies.

## 1 Introduction

**Figure 1:**
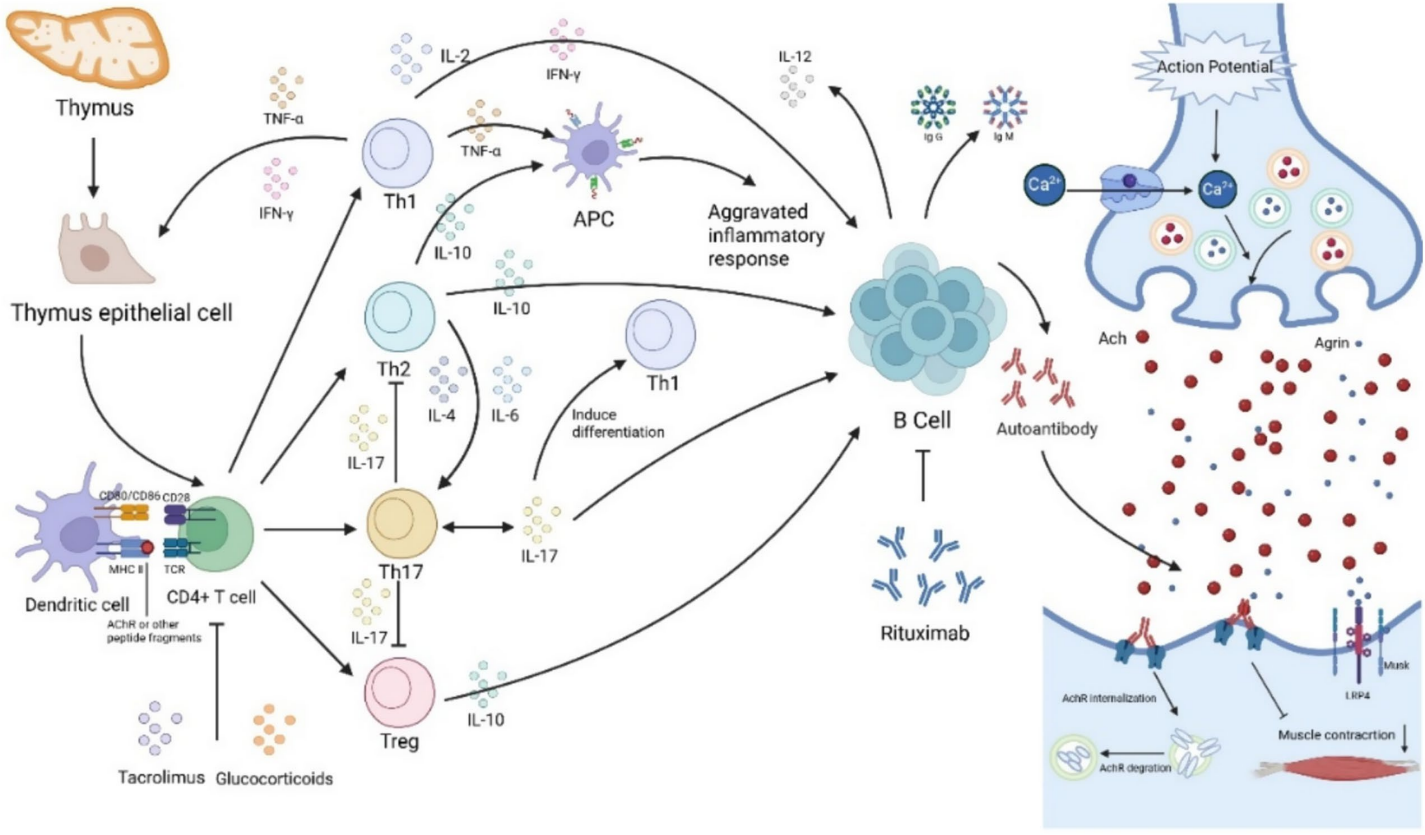
The role of cytokines in the onset of myasthenia gravis.

Myasthenia gravis (MG) is an autoimmune disease characterized by neuromuscular junction transmission disorders. It is clinically divided into ocular myasthenia gravis (OMG) and generalized myasthenia gravis (GMG). OMG accounts for about 15% of the total number of MG cases and about 85% of GMG (1–3). The pathological mechanism of MG involves complex immune system disorders, and thymus abnormalities are considered to be the initiation site that induces abnormal immune responses. In the thymus microenvironment, thymic epithelial cells and myolike cells express their own AchR antigen, dendritic cells, follicular helper T cells (Tfh) form an ectopic germinal center with B cells, driving the activation of autoreactive T/B cells and producing pathogenic antibodies to attack the postsynaptic membrane of the neuromuscular junction (4–6). The study found that under the action of different transcription factors, CD4 helper T cells (Th) can differentiate into different cell subpopulations, secrete proinflammatory and anti-inflammatory cytokines, and mediate the production process of anti-acetylcholine receptor (AchR) antibodies. The subpopulation of proinflammatory Th cells in MG peripheral blood increases, such as Th1 cells, Th2 cells, Th17 cells, and Tfh cells, while the inhibitory regulatory T cells (Tregs) are reduced, resulting in excessive activation of B cells to produce pathological autoantibodies. Th1 cells secrete cytokines such as Interleukin (IL)-2, IL-12, Interferon (IFN)-*γ*, Tumor Necrosis Factor (TNF)-*α*, and may participate in the expression of major histocompatibility complex II molecules, thereby upregulating the expression of AChR. Th2 cells secrete cytokines such as IL-4, IL-6, IL-10, IL-13, etc., and the effects of cytokines such as IL-4, IL-6, IL-10, and IL-13 are relatively complex and change according to different environments. On the one hand, anti-inflammatory cytokines secreted by Th2 cells can downregulate the functions of antigen-presenting cells and Th1 cells and reduce the immune response. On the other hand, they can also act as growth and differentiation factors of B lymphocytes, promoting the formation of antibody responses. Th17 cells are a newly discovered Th cells associated with IL-23. IL-23 can promote Th17 cells to differentiate and secrete IL-17. Th17 cytokines can participate in the antibody-mediated MG pathogenesis in the absence of Th1 cells (7, 8). Cytokine-mediated chronic inflammatory pathways are upregulated in disease progression, which are considered the core drivers for the promotion of inflammatory responses and tissue damage. In healthy individuals, these processes will be inhibited by Treg cells. Treg cells in MG patients are downregulated, resulting in uninhibited inflammation and B cell activation. Therefore, in the onset and pathological process of MG, both cellular and humoral immunity are regulated by Th cells and their secreted cytokine network (9, 10) (Figure 1). In addition, previous studies have shown that plasma replacement in patients with MG crisis can improve symptoms such as respiratory failure more quickly and effectively than intravenous immunoglobulin. It is further inferred that plasma replacement can effectively remove inflammatory factors in the blood through the filter membrane to achieve more effective treatment (1).

Treatment of MG includes non-immunosuppressive agents, such as cholinesterase inhibitors. Immunosuppressants such as corticosteroids, cyclosporin A, and tacrolimus (TAC), as well as other methods such as surgery and plasma replacement. In recent years, the treatment of MG has gradually shifted from traditional treatment to biological agent treatment (11). Among them, rituximab (RTX) specifically eliminates B cells by targeting the B cell membrane molecule CD20, becoming an important choice for antibodies to mediate autoimmune diseases. RTX monotherapy for newly diagnosed systemic AChR antibody-positive MG is considered effective and safe (12, 13). Although these drugs are widely used in clinical practice, their effects on plasma cytokine levels in patients with generalized MG (GMG) have not been systematically compared. Therefore, there is no unified standard for drug selection, and it depends more on the experience of clinicians. This study aims to characterize the associations between glucocorticoids (GC), TAC, and RTX and plasma cytokine profiles in patients with GMG, providing exploratory immunological insights that may inform future hypothesis-driven research.

## 2 Materials and methods

### 2.1 General clinical data

A retrospective collection of 65 cases of GMG hospitalized at the Department of Neurology, General Hospital of the People’s Liberation Army, from July 2022 to May 2024 was conducted. All enrolled patients met the diagnostic criteria of the “2020 Guidelines for the Diagnosis and Treatment of Myasthenia Gravis in China,” and the following exclusion criteria were applied: coexisting autoimmune diseases; prior exposure to intravenous immunoglobulin, plasma exchange, or any additional immunosuppressive agents; and the presence of acute or chronic infections or other inflammatory comorbidities. Based on the treatment regimens, the patients were divided into four groups: the non-medicated patients (NM) group, the GC group, the TAC group, and the RTX group. Additionally, 30 age- and sex-matched healthy individuals undergoing routine check-ups at the hospital during the same period were selected as a healthy control (HC) group, with inclusion criteria of healthy adults over 18 years of age, individuals with MG, thymoma, or other autoimmune diseases were excluded, as well as those with acute or chronic infections, chronic inflammatory disorders, endocrine diseases, malignancy, or those taking glucocorticoids, immunosuppressants, or other medications known to affect immune function. Clinical classification was assessed according to the recommendations of the Myasthenia Gravis Foundation of America (MGFA), and GMG patients underwent scoring using the Myasthenia Gravis Activities of Daily Living Scale (MG-ADL) and the Quantitative Myasthenia Gravis Score (QMG) (14). This study was conducted following the Helsinki Declaration and was approved by the Ethics Committee of the General Hospital of the People’s Liberation Army (HZKY-PJ-2022-22). Written informed consent was obtained from all participants.

### 2.2 Treatment

According to the treatment plan received by the patients, it was divided into four groups: the NM group, the GC group, the TAC group, and the RTX group. The inclusion of glucocorticoids, tacrolimus, and rituximab was based on their predominant use in our clinical practice during the study period and their distinct immunological mechanisms, which allowed them to serve as representative therapeutic strategies for comparing cytokine profiles under different classes of immunosuppression. All patients were in the maintenance treatment stage, and the treatment time of different treatment groups remained consistent. The use of glucocorticoids was performed using a small dose escalation method (the initial dose was 20–30 mg/d, with 5 mg per week increased until the daily dose reached 1 mg/kg, and then the treatment was maintained for 4–8 weeks). The dosage of tacrolimus for single immunotherapy was 3 mg/d, and the medication is uniformly taken orally on an empty stomach when you get up in the morning. The dose of rituximab was 375 mg/m^2^/week, and the treatment was administered continuously for 4 weeks.

In this study, “monotherapy” refers exclusively to the use of a single immunosuppressive agent (GC, TAC, or RTX). Pyridostigmine was administered as symptomatic therapy in all patient groups when clinically indicated, but it was not included in the definition of monotherapy because it does not exert immunomodulatory effects.

### 2.3 Cytokine levels

2 mL of peripheral venous blood was collected using EDTA anticoagulation tubes, and plasma was separated within 4 h (1,000 × g, 10 min). Cytokines were detected using a multiplex microsphere flow immunofluorescence luminescence method. 25ul of buffer solution, capture microsphere antibodies, detection antibodies, and plasma (or serially diluted calibrators) were sequentially added to flow tubes, incubated away from light at room temperature with shaking for 2 h (300 r/min); then, 25ul of phycoerythrin-labeled streptavidin was added and incubated for 30 min. 1 mL of diluted wash buffer (10x concentrated wash solution: distilled water = 1:9) was added to each tube, vortexed, and centrifuged at 300 × g for 5 min, discarding the supernatant; then, 120ul of diluted wash buffer was added to resuspend each tube, with flow cytometry detecting the fluorescence intensity of calibrator and sample tubes; the LEGENDplex v8.0 software was used to calibrate and analyze plasma cytokine levels. Cytokine concentrations were analyzed using the original values reported by the clinical laboratory. When the analyte concentration was extremely low—far below the detection limit—the instrument algorithm automatically reported the value as 0, and these values were retained as measured for statistical analysis.

All samples were processed under identical assay conditions, and non-parametric rank-based tests were applied, making the analysis robust to fluctuations in absolute quantification at low concentration levels.

### 2.4 Statistical methods

This study used R Studio v4.4.2 for statistical analysis and graphical visualization. The normality test of each cytokine variable was performed using the Shapiro–Wilk test, and the comparison Groups package was used to perform descriptive statistics and comparisons on different groups. The non-parametric test method was used for the comparison of continuous variables between groups, specifically the Kruskal-Wallis H test was used for overall differences in multiple groups, and the statistical significance was set at *p* < 0.05. If the overall difference is significant, a pairwise comparison was performed, and the Wilcoxon rank sum test (Mann–Whitney U test) was used, and statistically significant group pairs were automatically screened, and the ggplot2 package and the ggpubr package were used for visualization.

All statistical analyses were performed using raw cytokine concentrations (pg/mL). Z-score standardization was applied only for visualization purposes, not for statistical testing. For pairwise group comparisons of cytokines, Wilcoxon rank-sum tests were used and the Benjamini–Hochberg–adjusted *p* values are summarized in Supplementary Table 1.

## 3 Results

A total of 65 GMG patients were enrolled, including 31 males and 34 females, and they were divided into four treatment groups: NM (*n* = 22), GC (*n* = 17), TAC (*n* = 17), and RTX (*n* = 9). An additional 30 age- and sex-matched individuals served as the healthy control (HC) group. Across the four GMG groups, baseline characteristics—including age, gender, disease duration, MGFA class, antibody subtype, thymoma status, thymectomy history, QMG score, and MG-ADL score—showed no meaningful differences. These findings indicate that the treatment groups were comparable at baseline (Table 1).

**Table 1:** Baseline characteristics of MG patients and healthy control groups.

Variables	HC (*n* = 30)	NM (*n* = 22)	GC (*n* = 17)	TAC (*n* = 17)	RTX (*n* = 9)	*p*-value
Age-years (mean ± SD)	57.2 ± 15.1	59.3 ± 14.0	57.2 ± 17.4	58.4 ± 16.2	51.4 ± 11.2	0.82
Gender (%)						0.97
Female	13 (43.3%)	11 (50%)	8 (47.1%)	8 (47.1%)	4 (44.4%)	
Male	17 (56.7%)	11 (50%)	9 (52.9%)	9 (52.9%)	5 (55.6%)	
Disease duration, years	—	1.6 (1.0–2.3)	1.7 (1.1–2.4)	1.5 (0.9–2.2)	1.8 (1.2–2.5)	0.85
MGFA class, *n* (%)	—					
Class II	—	12 (54.6%)	9 (53.0%)	9 (53.0%)	4 (44.4%)	0.61
Class III	—	8 (36.3%)	5 (29.4%)	6 (35.2%)	4 (44.4%)	0.86
Class IV	—	2 (9.1%)	3 (17.6%)	2 (11.8%)	1 (11.1%)	0.83
Antibody subtype, *n* (%)	—					
AChR+	—	14 (63.6%)	10 (58.8%)	11 (64.7%)	4 (44.4%)	0.25
MuSK+	—	5 (22.7%)	4 (23.5%)	4 (23.5%)	3 (33.3%)	0.92
Seronegative	—	3 (13.6%)	3 (17.6%)	2 (11.8%)	1 (11.1%)	0.75
Thymoma, *n* (%)	—	3 (13.6%)	2 (11.8%)	2 (11.8%)	1 (11.1%)	0.80
Thymectomy, *n* (%)	—	6 (27.3%)	5 (29.4%)	4 (23.5%)	3 (33.3%)	0.77
Clinical assessments						
QMG	—	12.05 ± 6.21	13.41 ± 6.53	10.07 ± 4.80	13.00 ± 2.45	0.34
MG-ADL	—	8.05 ± 4.10	8.58 ± 5.24	9.00 ± 4.96	10.00 ± 3.82	0.74

HC, healthy control group; GMG, generalized myasthenia gravis; NM, non-medicated patients; GC, glucocorticoids; TAC, tacrolimus; RTX, rituximab; MG-ADL, myasthenia gravis daily life scale; QMG, quantitative score of myasthenia gravis; MGFA, Myasthenia Gravis Foundation of America; AchR, Acetylcholine Receptor; MuSK, Muscle-Specific Kinase. Percentages are calculated based on subgroup totals and rounded to one decimal place.

**Figure 2:**
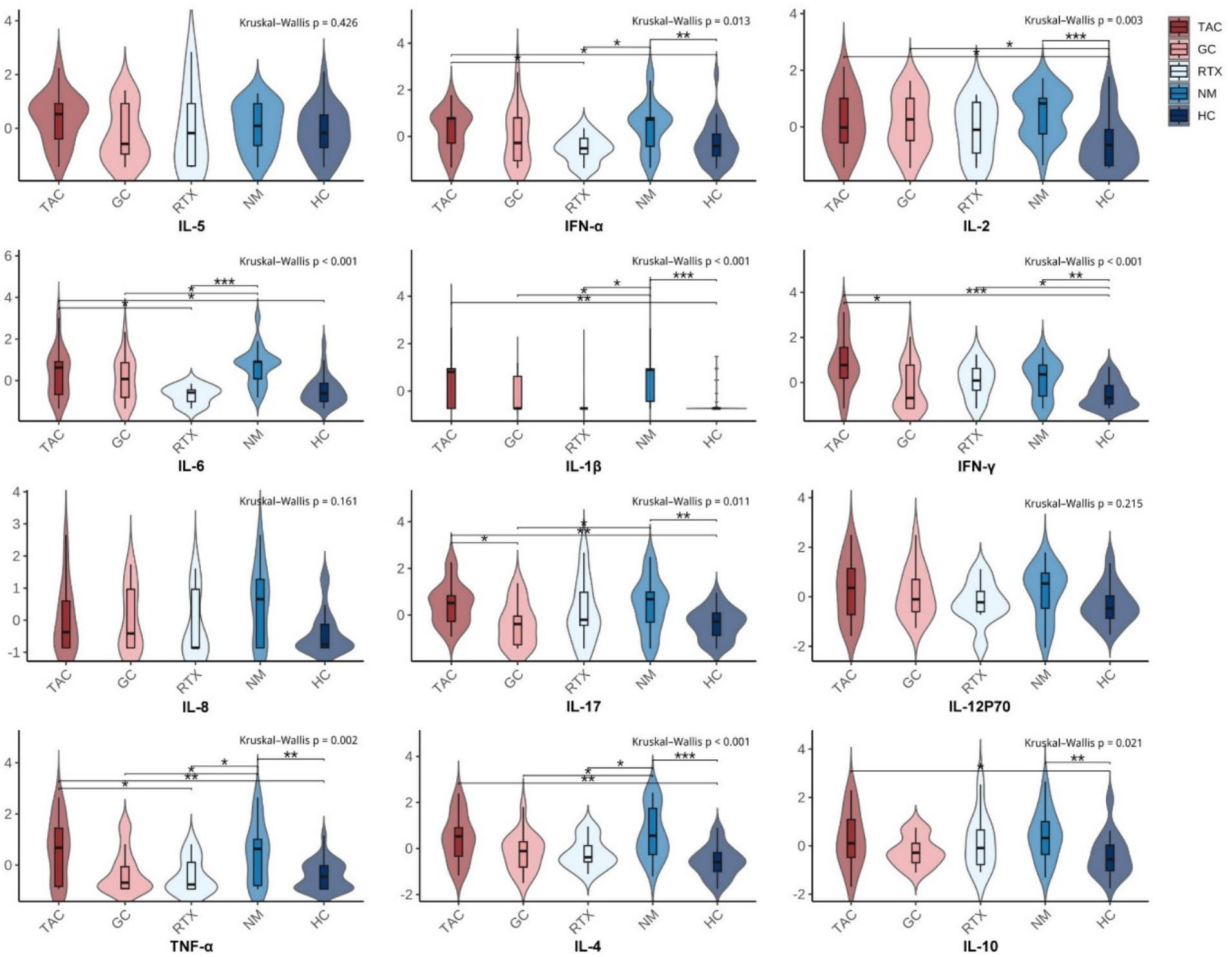
Distribution of cytokine levels across treatment groups with pairwise Wilcoxon comparisons. Each subplot presents the distribution of a specific cytokine (Z-score normalized) across five groups: TAC (tacrolimus), GC (glucocorticoids), RTX (rituximab), NM (non-medicated GMG patients), and HC (healthy controls). Violin plots display smoothed distributions overlaid with boxplots (median and interquartile range). The Kruskal–Wallis test was used to assess overall group differences (shown at top of each panel). Pairwise comparisons were performed using Wilcoxon rank-sum tests. Significance levels are indicated by asterisks: *p<0.05; **p<0.01; ***p<0.001.

Compared with the HC group, plasma levels of IL-1β, IL-2, IL-4, IL-10, IL-17, TNF-*α*, IFN-α, and IFN-*γ* were significantly higher in GMG patients in the NM group. These results indicate that GMG patients exhibited a significant immune activation state with proinflammatory cytokines at higher levels *in vivo* (Table 2, Figure 2).

**Table 2:** Comparison of cytokines between the healthy control group, the non-medicated patients group, the glucocorticoid group, the tacrolimus group, and the rituximab group.

Cytokines	TAC (*n* = 17)	GC (*n* = 17)	RTX (*n* = 9)	NM (*n* = 22)	HC (*n* = 30)	*H*	*p*
IFN-α	2.01 (1.01, 2.03)	1.02 (0.30, 2.03)	0.80 (0.57, 1.20)	1.96 (0.88, 2.03)	0.89 (0.49, 1.38)	14.32	0.013^*^
IFN-γ	3.02 (2.11, 4.25)	0.72 (0.00, 3.02)	1.94 (1.27, 2.79)	2.38 (0.86, 3.02)	0.74 (0.30, 1.59)	18.15	0.001^**^
IL-10	0.95 (0.65, 1.45)	0.75 (0.54, 0.95)	0.85 (0.50, 1.23)	1.06 (0.71, 1.40)	0.60 (0.37, 0.91)	11.77	0.021^*^
IL-12P70	1.21 (0.67, 1.60)	0.98 (0.73, 1.38)	0.92 (0.73, 1.14)	1.30 (0.80, 1.51)	0.80 (0.60, 1.05)	6.05	0.215
IL-17	1.74 (1.04, 2.02)	0.94 (0.15, 1.24)	1.10 (0.89, 2.15)	1.88 (1.01, 2.16)	1.02 (0.50, 1.36)	15.55	0.011^*^
IL-1β	2.28 (0.00, 2.48)	0.00 (0.00, 2.00)	0.00 (0.00, 0.00)	2.42 (0.44, 2.47)	0.00 (0.00, 0.01)	22.37	<0.001^***^
IL-2	0.90 (0.56, 1.55)	1.08 (0.61, 1.55)	0.85 (0.33, 1.46)	1.44 (0.76, 1.55)	0.51 (0.05, 0.86)	17.96	0.003^**^
IL-4	1.41 (0.90, 1.63)	1.03 (0.62, 1.27)	0.87 (0.75, 1.17)	1.42 (0.94, 2.13)	0.74 (0.50, 1.00)	23.22	<0.001^***^
IL-5	2.83 (1.50, 3.39)	1.24 (0.70, 3.39)	1.82 (0.07, 3.39)	2.20 (1.16, 3.39)	1.82 (1.05, 2.78)	4.99	0.426
IL-6	3.36 (1.16, 3.84)	2.43 (0.92, 3.76)	1.32 (0.58, 1.54)	3.84 (2.44, 3.84)	1.25 (0.59, 2.07)	25.84	<0.001^***^
IL-8	0.71 (0.00, 2.12)	0.65 (0.00, 2.65)	0.00 (0.00, 2.65)	2.20 (0.00, 3.10)	0.15 (0.00, 1.06)	6.65	0.161
TNF-α	3.79 (0.24, 5.56)	0.58 (0.05, 2.04)	0.41 (0.00, 2.44)	3.66 (0.32, 4.54)	1.14 (0.00, 2.14)	17.25	0.002^**^

Values are presented as median (Q1, Q3); all values represent raw cytokine concentrations in pg/mL. TAC, tacrolimus; GC, glucocorticoid; RTX, rituximab; NM, non-medicated patients group; HC, healthy control group. *p<0.05; **p<0.01; ***p<0.001. Detailed Benjamini–Hochberg–adjusted *p* values for all pairwise cytokine comparisons are provided in Supplementary Table 1.

Compared with the non-medicated group, the GC group showed lower plasma levels of IL-1β, IL-4, IL-6, IL-17, and TNF-*α*. The RTX group also showed lower levels of IL-1β, IL-4, IL-6, IFN-α, and TNF-α. In contrast, cytokine levels in the TAC group were closer to those observed in the non-medicated group (Figure 2). Overall, the three treatment groups displayed different cytokine distribution patterns, revealing heterogeneity in immune-inflammatory states among patients receiving GC, TAC, or RTX.

When comparing cytokine levels among the three treatment groups, GC, TAC, and RTX showed distinct patterns. The GC group had lower IL-17 and IFN-*γ* levels compared with the TAC group, while the RTX group showed lower IL-6, IFN-*α*, and TNF-α levels than the TAC group (Figure 2). These group-to-group differences outline the immunological diversity observed under different therapeutic regimens. The corresponding Benjamini–Hochberg–adjusted *p* values for these pairwise comparisons are summarized in Supplementary Table 1.

## 4 Discussion

As an autoimmune disorder involving T cells, B cells, complement activation, autoantibodies, and multiple cytokine pathways, myasthenia gravis (MG) is characterized by complex immune-inflammatory dysregulation (15). Imbalances between humoral and cellular immunity contribute to the increased expression of pro-inflammatory mediators during disease activity, which can exacerbate neuromuscular junction dysfunction and clinical symptoms (16). In this study, we observed distinct cytokine profile patterns among GMG patients receiving glucocorticoids, tacrolimus, or rituximab, reflecting heterogeneity in immune-inflammatory status across treatment groups.

Previous studies have shown that Th1 and Th17 subpopulations have significantly increased in the peripheral blood of MG patients, with increased levels of pro-inflammatory cytokines including IL-1β, IL-2, IL-6, and TNF-*α* (17). Our study found that the plasma levels of IL-1β, IL-2, IL-4, IL-10, IL-17, TNF-α, IFN-α, and IFN-*γ* cytokines in GMG patients who were not treated with drugs were significantly higher than those in healthy controls, and the elevated cytokines reflected the immune activation status of MG. In addition, we found that levels of inflammatory factors such as IL-4 and IL-10 were also significantly increased. IL-4 and IL-10 mainly control the intensity of the immune response by inhibiting the Th1/Th17 response and inducing Treg cell differentiation. In this study, elevated IL-4 and IL-10 suggest characteristics of an imbalanced immune response in patients with MG.

Because this was a retrospective study, obtaining pretreatment plasma samples from the same individuals was not feasible. Therefore, we included untreated GMG patients whose disease severity did not significantly differ from that of the treatment groups and compared cytokine levels across these categories. Within this cross-sectional framework, patients receiving glucocorticoids, tacrolimus, or rituximab exhibited differing cytokine distribution patterns. Glucocorticoids significantly reduced proinflammatory cytokines such as IL-1β, IL-6, IL-17, and TNF-*α*, and demonstrated a notable immunosuppressive effect; while rituximab showed a significant effect in regulating the levels of IL-1β, IL-6, IFN-α, and TNF-α. In contrast, no statistically significant differences were observed between the TAC group and the non-medicated group for most cytokines. In small samples, a lack of statistical significance does not imply an absence of immunomodulatory effects. Rather, this result is more likely related to the limited sample size. Previous studies have shown that tacrolimus can improve clinical symptoms in patients with MG, and the exact mechanism remains unclear, though it may involve one or more of its immunomodulatory functions, such as the regulation of ryanodine receptor/calcium release channels in the sarcoplasmic reticulum and the increase in the transport of glucocorticoid receptors from the sarcoplasmic reticulum to the nucleus, which may be responsible for the observed therapeutic effects (18, 19).

In our research results, the levels of proinflammatory cytokines of IL-1β, IL-6, IL-17, TNF-*α*, and IFN-α in patients treated with rituximab and glucocorticoids decreased significantly. Rituximab is a B-cell depletion agent. It reduces plasma cell and plasma blast production from upstream, thereby directly reducing the production of autoantibodies, interrupting the inflammatory cascade response, and reducing the production of proinflammatory factors. Glucocorticoids can inhibit inflammatory responses, change cytokines and lymphocyte dynamics, improve phagocytic function, interrupt the inflammatory cascade waterfall response, protect AChR from damage, and alleviate the disease. Related studies found that multiple injections of recombinant IL-1ra daily for 2 weeks significantly reduced the symptoms of the experimental autoimmune MG animal model (EAMG) and reduced plasma C3, anti-AChR IgG, IFN-*γ*, TNF-*α*, IL-1β, IL-2, and IL-6 levels. Anti-IL-6 antibody treatment significantly reduced anti-AChR antibody levels as well as downregulation of Th17 cell function (20). Further supports the importance of proinflammatory cytokines in EAMG induction. The study found that the expression of IL-1β and IL-6 in hyperplastic thymus tissues increased, and the mRNA and protein levels of IL-6 were upregulated in thymic epithelial cells. *In vitro* experiments, proliferative thymic epithelial cells from MG patients spontaneously generated a large amount of IL-1, while cells from thymoma spontaneously produced a small amount of IL-1. These studies show that IL-6 and IL-1 are important for T and B cell growth and differentiation, are involved in regulating local immune responses of the thymus, and may lead to common thymus hyperplasia in MG (21).

*In vivo* experiments, IL-6 can in turn promote Treg to differentiate into Th17, which is secreted by Th17 cells and is one of the main cytokines that cause tissue inflammation. IL-17 is also considered key to autoantibodies in the EAMG model, promoting elevated anti-AChR antibody production (22). At the onset of MG, TNF-*α* can promote the proliferation and differentiation of thymocytes, and can also induce the activation of antigen-presenting cells to produce related cytokines, which promote the proliferation and activation of T cells (23). It has been reported that the level of TNF-*α* and the number of TNF-mRNA expressed in MG patients are higher than those of normal people. The level of TNF-α in plasma is related to the severity of MG symptoms, and the level in GMG patients is higher than that in OMG patients. After immunomodulation treatment, its concentration decreases as MG symptoms improve (24). TNF-*α* is involved in the formation of germinal centers in the spleen and lymph nodes and the regulation of adhesion molecules on B and T cells. TNF-*α* may contribute to T and B cell activation and subsequent AChR antibody production in MG (25). There have been studies that have reported spontaneous neutralizing antibodies against IFN-α and IL-12 in thymoma-related autoimmune diseases (26). Additional studies suggest that autoantibodies against high titers of IFN-α2 are visible in thymoma-associated MG and thymoma without MG (27). Studies on the pathogenesis of IFN-α in MG are rare, and one study showed that IFN-α reduces EAMG occurrence by reducing anti-AchR IgG1 and IgG2 b levels as well as lymph node and spleen CD4^+^T cells (28). Therefore, the role of cytokines in the onset of MG is diverse and complex. Although some cytokines have more obvious roles in the onset of MG, the occurrence of the disease usually requires the synergy of multiple cytokines. IL-4 is one of the Th2 cytokines and plays an important role in the onset of MG. IL-4 is an effective growth and differentiation factor of B cells and stimulates class switching and autoantibody production. IL-4 also supports the inhibitory immune response elicited by Treg cells (29). These findings suggest a complex role for IL-4 in MG, potentially balancing both pro- and anti-inflammatory pathways. However, the reasons for the decline of IL-4 in the glucocorticoid and rituximide group still need further exploration (30).

Glucocorticoids suppress immune responses not only by regulating cytokines, but also clinically as rapid disease remission. As a core drug in MG treatment, it reduces the release of proinflammatory factors by inhibiting the NF-kB signaling pathway while enhancing Treg cell function, thus performing superiorly in acute phase symptom control (31). In addition, hormone therapy also exhibits a wide range of immunosuppressive effects, suitable for early management in most patients with MG. However, its long-term use may trigger glucocorticoid-related side effects, such as increased risk of infection and metabolic disorders (32). B-cell depletion agents are increasingly used in clinical practice, especially in the treatment of antibody-mediated autoimmune diseases. Unlike glucocorticoids, rituximab reduces plasma cells or plasmablasts that produce autoantibodies by specifically clearing B cells, blocking antibody production from the source, thereby indirectly affecting the cytokine network, significantly reducing the production of antibodies and the formation of immune complexes (33). This study further demonstrates that in the comparison of different treatment options, glucocorticoids and B cell depletion agents significantly inhibited the increase of proinflammatory cytokines and played an immunosuppressive role by inhibiting proinflammatory cytokines. Tacrolimus exerts relatively modest immunomodulatory effects, mainly regulating the immune response by inhibiting the proliferation and activation of T cells. Although its inhibitory effect on IL-6, IL-17, TNF-*α*, and IFN-α is not as significant as that of glucocorticoids and B-cell depletion agents, tacrolimus maintains immune tolerance by reducing the production of cytokines and activation of immune cells. It is more effective in maintenance therapy and is suitable for patients with slow disease progression or stable stages, especially those who refuse to receive steroid therapy or contraindicate other immunosuppressants, and has a good effect on long-term control of disease activity (34).

The powerful immunosuppressive effect of glucocorticoids is suitable for acute attacks, but long-term use may lead to a series of side effects; tacrolimus is suitable for patients with MG who cannot tolerate the side effects of glucocorticoids and other immunosuppressants or have poor efficacy, especially those with RyR antibody positive. The main side effects include increased blood sugar, decreased blood magnesium, tremor, liver and renal function damage, and rare myelosuppression. As a targeted biological agent, RTX efficiently removes CD20^+^B cells. The main side effects include fever, chills, bronchospasm, leukopenia, thrombocytopenia (35). The three treatments have their own advantages and disadvantages, and how to choose the individualized treatment has become a difficult point. The cytokine profile differences observed across treatment groups suggest that GMG may present with distinct underlying immunoinflammatory states under different immunosuppressive backgrounds. These observational findings provide preliminary clues for further investigations into the immunological action patterns and potential pharmacological mechanisms of these therapeutic agents.

This study has several limitations. First, it was an observational, retrospective, and non-randomized study, making it difficult to fully rule out potential confounding factors, such as subtle differences in disease activity, socioeconomic influences, and other unmeasured variables. Second, the overall sample size was relatively small, and some treatment groups—particularly the RTX group—had limited numbers of patients, which reduced the statistical power for several comparisons. Third, cytokine measurements were obtained from cross-sectional samples, and standardized paired samples before and after treatment were not available from the same individuals. As a result, temporal changes in cytokine levels within patients could not be assessed, nor could their relationship with immune-cell subset dynamics or clinical outcomes be clearly determined. Additionally, the study did not include clinical outcome data, preventing assessment of whether cytokine levels correlated with symptomatic improvement. In addition, this study was conducted at a single center in China, where treatment decisions may be influenced by local guidelines, drug availability, and patterns of clinical practice, which may limit the generalizability of the findings. Future multicenter, prospective studies with longitudinal sampling and clinical outcome measures are needed to confirm these findings, and the present results should be regarded as hypothesis-generating.

## 5 Conclusion

This study compared plasma cytokine levels in GMG patients treated with glucocorticoids, tacrolimus, or rituximab. The three treatment groups showed noticeable differences in several inflammatory cytokines. These findings offer a clearer picture of cytokine variation in GMG under commonly used treatment regimens and provide reference data for understanding immune activity in this condition. Future multicenter, prospective studies incorporating longitudinal cytokine monitoring, immune-cell profiling, and standardized clinical outcome assessments are needed to further elucidate the immunological characteristics of GMG under different treatment regimens.

## Data Availability

The original contributions presented in the study are included in the article/Supplementary material, further inquiries can be directed to the corresponding authors.
