# Supplementary material for: Alterations in plasma cytokine profiles in generalized myasthenia gravis following different immunotherapeutic regimens
Source: Front Neurol. 2025 Dec 5;16:1728767. doi: 10.3389/fneur.2025.1728767 (PMC12714632; doi:10.3389/fneur.2025.1728767)
Supplement: Supplementary file 1 [file Table_1.docx]

**Supplementary Table 1.** BH-adjusted P values for pairwise comparisons of cytokines

| Variable | GC vs TAC | HC vs GC | HC vs NM | HC vs RTX | HC vs TAC | NM vs GC | NM vs RTX | NM vs TAC | RTX vs GC | RTX vs TAC |
| --- | --- | --- | --- | --- | --- | --- | --- | --- | --- | --- |
| IFN-α | 0.240 | 0.506 | **0.005** | 0.548 | **0.014** | 0.205 | **0.012** | 0.954 | 0.318 | **0.027** |
| IFN-γ | **0.016** | 0.688 | **0.003** | **0.040** | **0.000** | 0.225 | 0.647 | 0.100 | 0.498 | 0.089 |
| IL-10 | 0.185 | 0.245 | **0.003** | 0.117 | **0.039** | 0.050 | 0.557 | 0.692 | 0.434 | 0.788 |
| IL-12P70 | 0.783 | 0.156 | 0.068 | 0.714 | 0.106 | 0.713 | 0.231 | 0.921 | 0.483 | 0.332 |
| IL-17 | **0.017** | 0.650 | **0.008** | 0.505 | **0.003** | **0.030** | 0.542 | 0.955 | 0.400 | 0.482 |
| IL-1β | 0.056 | 0.142 | **0.000** | 0.948 | **0.002** | **0.017** | **0.029** | 0.699 | 0.479 | 0.050 |
| IL-2 | 0.972 | **0.022** | **0.000** | 0.292 | **0.012** | 0.237 | 0.138 | 0.355 | 0.517 | 0.434 |
| IL-4 | 0.058 | 0.090 | **0.000** | 0.188 | **0.002** | **0.020** | **0.045** | 0.609 | 0.936 | 0.137 |
| IL-5 | 0.137 | 0.626 | 0.409 | 0.841 | 0.088 | 0.371 | 0.484 | 0.399 | 0.957 | 0.329 |
| IL-6 | 0.458 | 0.121 | 0.086 | 0.560 | **0.015** | **0.046** | **0.000** | 0.277 | 0.106 | **0.035** |
| IL-8 | 1.000 | 0.350 | 0.077 | 0.542 | 0.252 | 0.282 | 0.153 | 0.224 | 0.436 | 0.394 |
| TNF-α | 0.057 | 0.711 | **0.001** | 0.851 | **0.003** | **0.036** | **0.021** | 0.910 | 0.529 | **0.029** |

**Note:** Wilcoxon rank-sum tests were used for pairwise group comparisons of cytokine concentrations. P values were adjusted using the Benjamín–Hochberg method.
